# Astaxanthin Exerts Anabolic Effects via Pleiotropic Modulation of the Excitable Tissue

**DOI:** 10.3390/ijms23020917

**Published:** 2022-01-14

**Authors:** Mónika Gönczi, Andrea Csemer, László Szabó, Mónika Sztretye, János Fodor, Krisztina Pocsai, Kálmán Szenthe, Anikó Keller-Pintér, Zoltán Márton Köhler, Péter Nánási, Norbert Szentandrássy, Balázs Pál, László Csernoch

**Affiliations:** 1 Department of Physiology, Faculty of Medicine, University of Debrecen, 4012 Debrecen, Hungary; gonczi.monika@med.unideb.hu (M.G.); csemer.andrea@med.unideb.hu (A.C.); szabo.laszlo@med.unideb.hu (L.S.); sztretye.monika@med.unideb.hu (M.S.); fodor.janos@med.unideb.hu (J.F.); deak-pocsai.krisztina@med.unideb.hu (K.P.); nanasi.peter@med.unideb.hu (P.N.); szentandrassy.norbert@med.unideb.hu (N.S.); csl@edu.unideb.hu (L.C.); 2 Doctoral School of Molecular Medicine, University of Debrecen, 4012 Debrecen, Hungary; 3 Carlsbad Research Organization Ltd., 9244 Újrónafő, Hungary; kszenthe@rt-europe.org; 4 Department of Biochemistry, Albert Szent-Györgyi Medical School, University of Szeged, 6720 Szeged, Hungary; keller.aniko@med.u-szeged.hu (A.K.-P.); kohler.zoltan@med.u-szeged.hu (Z.M.K.); 5 Department of Dental Physiology and Pharmacology, Faculty of Dentistry, University of Debrecen, 4012 Debrecen, Hungary; 6 Department of Basic Medical Sciences, Faculty of Dentistry, University of Debrecen, 4012 Debrecen, Hungary

**Keywords:** astaxanthin, metabolism, food intake, gene expression, skeletal muscle, cardiac action potential, arcuate nucleus, excitability, inhibitory postsynaptic current

## Abstract

Astaxanthin is a lipid-soluble carotenoid influencing lipid metabolism, body weight, and insulin sensitivity. We provide a systematic analysis of acute and chronic effects of astaxanthin on different organs. Changes by chronic astaxanthin feeding were analyzed on general metabolism, expression of regulatory proteins in the skeletal muscle, as well as changes of excitation and synaptic activity in the hypothalamic arcuate nucleus of mice. Acute responses were also tested on canine cardiac muscle and different neuronal populations of the hypothalamic arcuate nucleus in mice. Dietary astaxanthin significantly increased food intake. It also increased protein levels affecting glucose metabolism and fatty acid biosynthesis in skeletal muscle. Inhibitory inputs innervating neurons of the arcuate nucleus regulating metabolism and food intake were strengthened by both acute and chronic astaxanthin treatment. Astaxanthin moderately shortened cardiac action potentials, depressed their plateau potential, and reduced the maximal rate of depolarization. Based on its complex actions on metabolism and food intake, our data support the previous findings that astaxanthin is suitable for supplementing the diet of patients with disturbances in energy homeostasis.

## 1. Introduction

Astaxanthin (AX) is a safe, lipid-soluble, bioavailable natural carotenoid compound present in several microorganisms and various aquatic and nonaquatic species including crustaceans, fish or the flamingo. A major source of AX is *Haematococcus pluvialis*, a green microalga with high AX content [1,2,3,4]. AX is a potent nutraceutical widely used as a nutritional supplement with antioxidant and anticancer actions. AX was shown to prevent diabetes, cardiovascular diseases, neurodegenerative disorders, and stimulate immunization [5,6]. As an antioxidant, AX reduces oxidative stress, increases the bioavailability of nitric oxide (NO) and the activity of antioxidant enzymes, and maintains the rheological properties of the blood [7]. It ameliorates skeletal muscle atrophy, and it exerts neuroprotective actions [8,9]. AX’s anti-inflammatory properties involve modulating the NF-κB and MAPK signaling pathways, reducing the release of pro-inflammatory cytokines and increasing reverse cholesterol transport by HDL [10].

Furthermore, AX is believed to have an important role in the whole-body metabolism and helps to keep the body weight in the normal range [11,12]. Nevertheless, studies have shown that AX accelerates lipid breakdown during physical exercise, decreases body fat, and promotes muscle work during exercise [13,14,15].

The molecular mechanisms underlying adequate skeletal muscle maintenance involve an interplay between multiple signaling pathways. Under physiological conditions, a network of interconnected signals serve to control the precise balance between muscle protein synthesis and proteolysis. It includes changes in mTOR signaling stimulating protein synthesis [16,17], improvement of insulin sensitivity, and increased glucose uptake and breakdown with the consequential reduction of hyperglycemia [18,19,20]. Fatty acid metabolism is also affected by de novo synthesis of essential lipids for the formation of cell membrane, the production of extra energy via beta-oxidation, and lipid modification of proteins [21,22,23,24,25].

It is well established that the hypothalamus has a great impact on regulation of energy homeostasis. Neurons of the hypothalamic arcuate nucleus are influenced by peripheral signals and levels of nutrient molecules and affect food intake differently. The proopiomelanocortin (POMC) neurons inhibit while the neuropeptide Y (NPY) and agouti-related peptide (AgRP) positive ones increase food intake [26,27,28]. The latter ones also provide GABAergic inhibition on POMC neurons [29].

In the present work, we aimed to analyze the effects of AX on food intake and body weight changes. Chronic AX dietary supplementation increased food intake and led to increased body weight. This increased food intake is at least partially due to the enhanced inhibition of POMC neurons inhibiting this process. Our data support the hypothesis that glucose metabolism is affected by AX in a way which exerts anti-diabetic actions but partially challenges the view on findings with altered food intake.

## 2. Results

### 2.1. Actions of Astaxanthin Feeding on Metabolism and Food Intake

**Figure 1:**
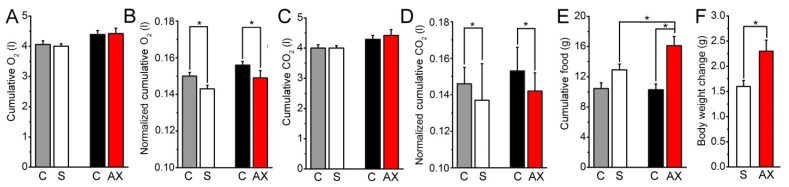
Metabolic effects of chronic astaxanthin feeding. (**A**,**B**) Actions on O_2_ consumption without (**A**) and with normalization on body weight (**B**). Gray column: control group before sham feeding (**C**); hollow column: control group after sham feeding (S); black column: AX fed group before feeding of astaxanthin-containing chow (**C**); red column: AX fed group after feeding of astaxanthin-containing chow (AX). All panels have the same arrangement as above; (**C**,**D**) Effects on CO_2_ consumption without (**C**) and with normalization on body weight (**D**); (**E**) Normalized actions on food intake; (**F**) Changes in body weight gain. * denotes significant differences at *p* < 0.05.

At first, the basic metabolic changes of control and AX fed mice were determined. Cumulative O_2_ usage was similar before and after the four weeks of special feeding both in control and AX group (Figure 1A); however, when these parameters were normalized to the actual body weight, a significant decrease was observed following the treatment in both groups (Figure 1B). There was no significant difference between O_2_ usage of control and AX groups. Similar observation was done regarding cumulative CO_2_ production (Figure 1C), and its normalized values (Figure 1D). More specifically, average cumulative food intake revealed to be significantly higher in AX group compared to its control counterpart following 4 weeks of special feeding. Furthermore, food intake increased significantly after the treatment within the AX group (Figure 1E), while this parameter was similar in control animals before and after 4 weeks of feeding. Altogether, the average body weight changes in the AX group were significantly higher as compared to the control group (Figure 1F).

Activity (X_amb_ and X_total_) was significantly reduced in both control and AX groups after 4 weeks of feeding. Other parameters involved in the overall metabolism measured in vivo were either lower (VO_2_ and VCO_2_), higher (cumulative water intake), or similar (RER, heat production, sleeping time) when comparing the data of control and AX animals before and after the 4 weeks of feeding. However, one must note that overall significant alterations were not observed as a consequence of the effect of AX administration (Table S1). Time-dependent changes of all the aforementioned parameters are plotted and presented in Figure S1A–F. Data from four individual measured points (equal with 72 min) were averaged in all animals within the same groups and were plotted as a function of time.

Using the respiratory ratio (VCO_2_/VO_2_), we calculated the calorific value (CV) using the equation (CV = 3.815 × 1.232 × RER), then total energy expenditure (TEE) was determined by multiplying CV and VO_2_. Subtracting resting/basal metabolic rate (RMR) from TEE we got the activity related energy expenditure (TEE_activity_). Both versions of energy expenditure are plotted as a function of time (Figure S1G,H, respectively).

In summary, mice kept on AX supplemented diet had increased food intake compared to the control group. In line with this, parameters related to in vivo metabolic activity did not change following the four weeks of special AX feeding.

### 2.2. Astaxanthin Feeding Influences the Activation of Signaling Molecules Affecting Skeletal Muscle Metabolism

**Figure 2:**
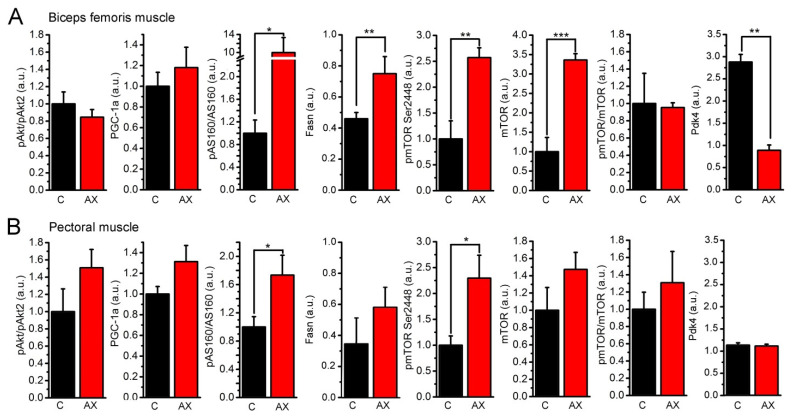
Effects of astaxanthin feeding on the levels of signaling proteins in biceps femoris and pectoral muscles. (**A**) Statistical analysis of changes in signaling protein expression of the biceps femoris muscle; (**B**) statistical analysis of changes in signaling protein expression of the pectoral muscles. Black columns (C): control, red columns (AX): astaxanthin feeding. Quantification of the results is reported as average ± SEM (*n* = 3–5); * *p* < 0.05, ** *p* < 0.01, *** *p* < 0.001.

Skeletal muscles are responsible for a significant proportion of metabolism of the whole organism; therefore, we aimed to investigate whether proteins regulating skeletal muscle metabolism are affected by the AX diet. AX was shown to accumulate in skeletal muscle [30]; therefore, the effects of long-term AX administration on representative muscles known to be involved in force production such as the hindlimb (biceps femoris muscle) or forelimb (pectoral muscle; Figure 2 and Figure S2) muscles of the mice were studied. The levels and phosphorylation of proteins involved in glucose uptake, regulation of cellular energy level, protein synthesis, and cell survival were also analyzed.

Nanostring nSolver analysis revealed significant differences in fatty acid synthase (Fasn) and stearoyl-CoA desaturase (Scd1) gene expression (2.47- and 2.06-fold change following AX-treatment, respectively, Figure S3), while pyruvate dehydrogenase kinase 4 (Pdk4) and xanthine dehydrogenase (Xdh) gene expression levels were lower (0.54-fold change and 0.66-fold change, respectively) in AX samples compared to control. Quantile normalization and class comparison generated a heatmap (Figure S3A) showing changes in gene expression between the two groups, but significant changes were also obvious following normalization of data for housekeeping genes (Figure S3B).

To evaluate the aforementioned changes at protein expression level, additional experiments were conducted using protein samples from biceps femoris, pectoral, and EDL muscles from control and AX fed animals. Similar alterations of Pdk4 protein expression as seen on RNA level were observed in all investigated muscle types, which is significantly lower in AX-treated biceps femoris and EDL samples as compared to control (Figure 2A,B and Figure S2C,D), respectively, while the Pdk4 protein expression showed only a slight decrease in the pectoral muscle of AX mice as compared with its control counterpart (Figure 2B and Figure S2B). Fasn protein expression was found to be significantly higher in AX-treated biceps femoris (Figure 2A and Figure S2A); however, it was significantly lower in AX-treated EDL samples (Figure S1C), while in pectoral muscles there was no significant difference between values from control and AX fed mice (Figure 2B and Figure S2B). We could not detect significant alteration of Scd1 protein level in muscle samples.

The levels and phosphorylation of proteins involved in glucose uptake, regulation of cellular energy level, protein synthesis, and cell survival were also analyzed. AX feeding increased mTOR phosphorylation (Ser2448) in biceps femoris and pectoral muscles (Figure 2A,B and Figure S2A,B). Since the total amount of mTOR also increased, the mTOR(pSer2448)/mTOR ratio did not change significantly following AX treatment. AX feeding significantly increased pAS160/AS160 levels in both biceps femoris and pectoral muscle samples (Figure 2A,B and Figure S2A,B). The expression of PGC-1α was slightly increased in biceps femoris and pectoral muscle samples following AX administration (Figure 2A,B and Figure S2A,B). Akt2 is the skeletal muscle specific isoform of Akt, and the p(Ser474)Akt2/Akt2 ratio did not change significantly in the studied samples, as only a mild elevation could be observed in pectoral muscles (Figure 2A,B and Figure S2A,B).

In summary, our findings regarding certain changes in enzymes of the skeletal muscles point towards higher levels of metabolism as well as anti-diabetic effects exerted by AX administration.

### 2.3. Hypothalamic Actions of Chronic Astaxanthin Feeding

AX fed animals had an increased food intake which raised the possibility that brain structures regulating food intake might be affected. As the hypothalamic arcuate nucleus has a key role in food intake, this nucleus became the subject of the next sets of experiments. Hypothalamic slices containing the arcuate nucleus were prepared and 17 neurons were patched from control and 15 from the AX fed mice.

**Figure 3:**
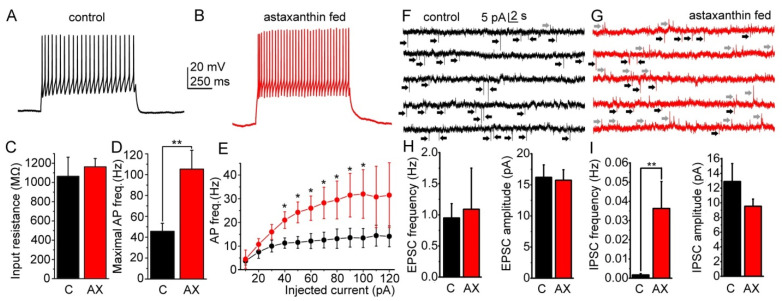
Effects of astaxanthin feeding on the excitability and synaptic currents of neurons in the hypothalamic arcuate nucleus. (**A**,**B**) Representative voltage traces of randomly chosen neurons of the arcuate nucleus in the control group (**A**, black) and in the AX fed population (**B**, red) elicited with 120 pA depolarizing current injection. (**C**–**E**) Statistical analysis of changes in input resistance (**C**), maximal action potential firing rate (**D**) and average action potential firing rate at different levels of depolarization (**E**). Black columns and graphs: control group (C), red columns, and graphs: astaxanthin fed population (AX). (**F**,**G**) Excitatory (black arrows) and inhibitory (gray arrows) postsynaptic currents (EPSCs and IPSCs, respectively) recorded from the control group (**F**, black) and from the AX fed group (**G**, red). (**H**,**I**) Statistical analysis of the EPSC frequency and amplitude (**H**) and the IPSC frequency and amplitude (**I**). Black columns: control group (C), red columns: astaxanthin fed population (AX). * *p* < 0.05, ** *p* < 0.01.

At first, the general changes of the excitability were checked (Figure 3A–E). There was no significant alteration of the input resistance (1065 ± 200 MΩ for control and 1163 ± 87 MΩ for fed mice, *p* = 0.36; Figure 3C). The maximal action potential firing frequency (measured as the frequency of the first two action potentials of the train elicited with 120 pA depolarization) was significantly greater (46 ± 8 Hz in control and 106 ± 18 Hz after feeding; *p* = 0.003; Figure 3D). The average firing rate elicited by increasing depolarizing steps also showed a tendency of increase with AX feeding (11.6 ± 2.5 vs. 24.3 ± 4.4 Hz at 50 pA depolarizing step, *p* = 0.01; 13.4 ± 4 vs. 32 ± 13 Hz at 100 pA depolarizing step, *p* = 0.0037; Figure 3E).

Secondly, changes of spontaneous excitatory and inhibitory postsynaptic potentials (EPSCs and IPSCs) were evaluated (Figure 3F–I). We found that AX feeding did not alter the EPSC frequency and amplitude (the frequency was 0.95 ± 0.23 Hz in control and 1.09 ± 0.66 Hz after feeding, *p* = 0.41; the amplitude was 16.24 ± 2.02 pA in control and 15.74 ± 1.67 pA after feeding, *p* = 0.42; Figure 3H). On the contrary, the IPSC frequency was very low in the control group (0.0017 ± 0.0007 Hz) which became much greater in the AX fed population (0.036 ± 0.014; *p* = 0.003). The IPSC amplitude did not change significantly (12.9 ± 2.4 pA in control and 9.53 ± 0.98 with AX feeding; *p* = 0.099; Figure 3I).

In summary, AX feeding led to increased excitability in neurons of the arcuate nucleus, together with an increased weight of inhibitory inputs.

### 2.4. Hypothalamic Effects of Acute Astaxanthin Application

**Figure 4:**
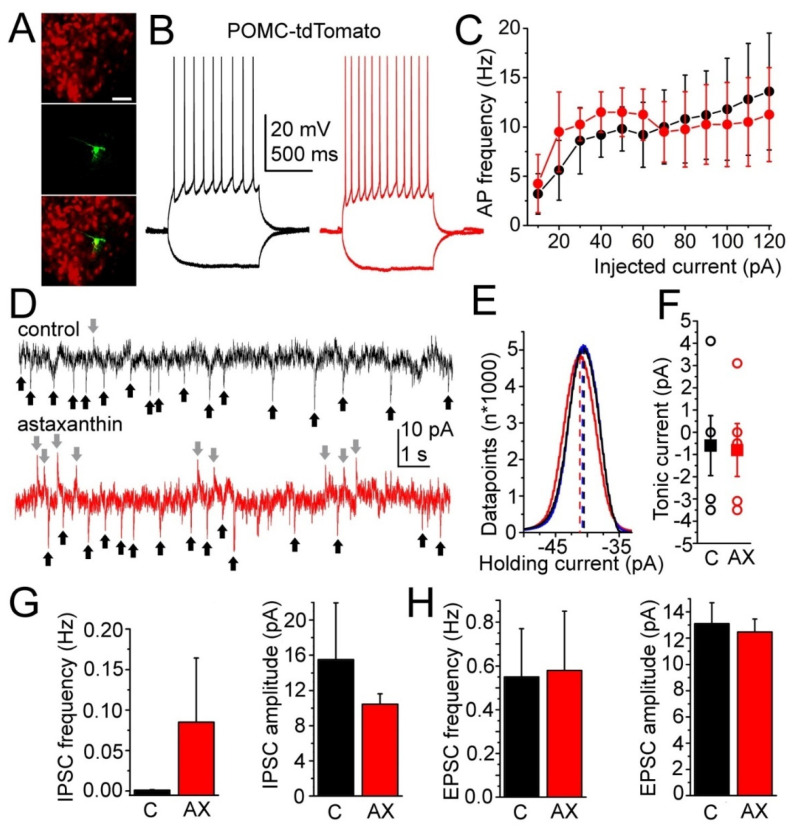
Changes by acute astaxanthin feeding on POMC-positive neurons of the arcuate nucleus. (**A**) Evaluation of the neuronal type. Upper panel: POMC-driven tdTomato expression in red. Middle panel: biocytin labeling (green). Lower panel: merged image. Images represent a single confocal z-stack with 1-µm thickness. Scale bar: 50 µm; (**B**) voltage traces from a POMC-positive neuron under control conditions (black) and with acute 2.5 µM AX treatment (red) elicited with −30 and +120 pA current injections; (**C**) comparison of the average firing rate with different depolarizing steps under control conditions and during AX application (red); (**D**) synaptic currents of a POMC-positive neuron under control conditions (above) and during AX treatment (below). Black arrows: EPSCs, gray arrows: IPSCs; (**E**) a representative trace of histogram analysis of the holding current under control (black) and in the presence of astaxanthin (red); (**F**) statistical comparison of the spontaneous fluctuation of the holding current (C, black) and the effect of astaxanthin on the holding current (AX, red; hollow circles: individual data, filled squares: average ± SEM); (**G**,**H**) statistical analysis of the IPSC (**G**) and EPSC (**H**) frequency and amplitude. Black columns: control conditions (C), red columns: astaxanthin treatment (AX).

In the next experiments, the acute responses to AX on different neuronal groups of the arcuate nucleus were evaluated. First, excitability and postsynaptic events of the POMC neurons (*n* = 8) were checked (Figure 4A). The input resistance, the maximal and average firing rates did not show significant differences with AX application compared to control (input resistance: 515.5 ± 59.74 MΩ in control and 667.33 ± 112.2 MΩ with AX, *p* = 0.12; maximal firing rate: 42.68 ± 9.63 Hz in control and 42.85 ± 10.69 Hz with AX, *p* = 0.45; firing rate at 50 pA depolarizing step: 9.8 ± 2.25 Hz in control and 11.5 ± 2.46 Hz with AX, *p* = 0.31; firing rate at 100 pA depolarizing step: 11.8 ± 5.18 Hz in control and 10.25 ± 4.07 Hz with AX, *p* = 0.41; Figure 4B,C). Tonic currents developed during AX application were also investigated. At −60 mV holding potential, the spontaneous fluctuation of the holding current was −0.6 ± 1.35 pA, whereas AX application elicited -0.8 ± 1.19 pA tonic currents. Altogether the tonic current elicited by AX was not significantly different from the spontaneous fluctuation (*p* = 0.46; Figure 4D–F).

Similar to the feeding experiments, there was no change in EPSC frequency and amplitude following acute AX application (the frequency was 0.55 ± 0.21 Hz in control and 0.58 ± 0.27 with AX, *p* = 0.46; the amplitude was 13.07 ± 1.57 pA in control and 12.46 ± 1.05 pA with AX, *p* = 0.37, Figure 4H). The IPSC frequency was increased with AX application (from 0.001 ± 0.0006 to 0.086 ± 0.079 Hz, Figure 4G). In a single case, when no IPSCs were seen in control, IPSCs occurred in AX with a frequency of 0.402 Hz; whereas a milder increase was seen in 4 other cases. The rest of the neurons (*n* = 3) lacked IPSCs in all experimental conditions. Because of this fluctuation in the strength of this effect, the difference between the two datasets was not significant (*p* = 0.158). The IPSC amplitude did not change significantly (15.55 ± 6.45 pA in control and 10.45 ± 1.19 pA with AX, *p* = 0.19; Figure 4G).

In summary, acute application of AX causes a tendency of increase in the frequency of inhibitory event on POMC neurons. In a subset of neurons, this effect might be stronger.

**Figure 5:**
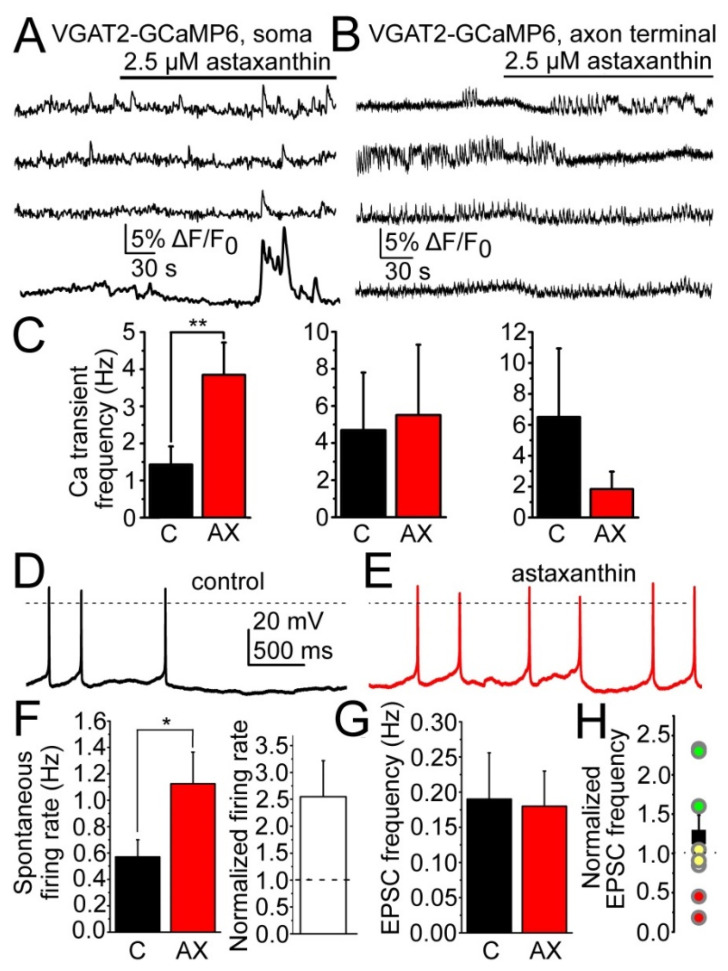
Changes in the excitability of the synaptic currents of the GABAergic inhibitory neurons of the arcuate nucleus. (**A**,**B**) Representative average calcium imaging traces from four GABAergic somata under control conditions and with application of 2.5 µM AX. (**C**) Statistical comparison of the calcium transient frequency of the GABAergic somata in control and with AX (black, C: control, red, AX) in the neuronal groups where increase, no change or decrease was seen (from left to right, respectively). Increase was seen in 77.3%, no change was observed in 9.1% and decrease was detected in 13.6% of the neuronal somata; (**D**,**E**) spontaneous activity of GABAergic neurons in control (black) and with AX (red); (**F**) statistical comparison of the spontaneous and normalized firing rate under control conditions and with AX (black, C: control, red, AX); (**G**) statistical representation of EPSC frequency in control (black, C) and with astaxanthin (red, AX); (**H**) average frequency change (black square) and frequency changes of the individual cases (green: increase, red: decrease, yellow: no change). * *p* < 0.05, ** *p* < 0.01.

Following these, the acute effects of AX on the local GABAergic neurons and synapses were investigated. For this, samples expressing GCaMP6f genetically encoded calcium indicator in GABAergic neurons (see Methods) were used. In total, 22 greater regions of interest (neuronal somata) produced calcium transients. Acute application of AX increased the calcium transient frequency or elicited calcium transients in 77.3% of the regions, it did not affect the activity in 9.1% and it decreased or blocked the calcium transient activity in the rest (13.6%; Figure 5A–C). Similar to this, smaller regions of interest (likely GABAergic axon terminals) produced similar response following AX administration. From 8 regions of interest, 3 decreased or ceased calcium transient activity, whereas 5 of them had increased calcium transient activity. The average calcium transient frequency was 2.42 ± 0.78 Hz which increased to 3.73 ± 0.75 Hz with AX; showing overall a 3.27- ± 1.57-fold increase. However, due to the decrease of calcium transient frequency in a smaller population, the calcium transient frequency was not significantly greater following acute AX treatment (*p* = 0.11; Figure 5C). Spontaneous action potential firing rate of GABAergic neurons increased from 0.57 ± 0.13 to 1.12 ± 0.24 Hz (2.55 ± 0.67-fold increase, *p* = 0.036, *n* = 10, Figure 5D–F). Potential changes of excitatory inputs were also checked and no statistical difference was found in EPSC frequency and amplitude of GABAergic neurons (frequency in control: 0.19 ± 0.06 Hz, frequency in AX: 0.18 ± 0.05 Hz, *p* = 0.44; amplitude in control: 12.54 ± 1.45 pA, amplitude in AX: 13.12 ± 1.55 pA, *p* = 0.38, *n* = 8, Figure 5G,H). In 3 cases, the EPSC frequency had 1.62.3–fold increase, but the change in the EPSC amplitude was only 1.21- ± 0.28-fold (Figure 5H).

Based on the data above, we hypothesize that AX directly increases the excitability of a GABAergic neuronal subpopulation in the arcuate nucleus; however, the increase in excitatory drive on certain GABAergic neurons cannot be excluded.

In conclusion, suppression on POMC neuronal population and the stimulation on GABAergic neurons are in accordance with the findings on increased food intake.

### 2.5. Effects of Astaxanthin on Action Potential Configuration in Canine Left Ventricular Myocytes

We were also interested to investigate whether AX diet could potentially exert arrhythmogenic actions thus we evaluated if cardiac excitability is affected in any way by AX supplementation.

**Figure 6:**
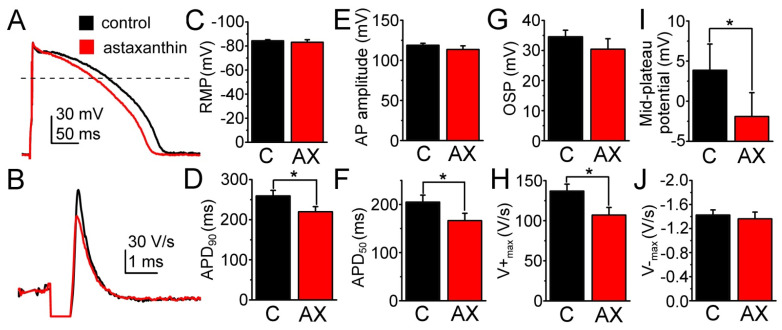
Effects of astaxanthin on the parameters of action potentials in canine left ventricular myocytes. (**A**) Representative superimposed action potentials recorded in control (black) and in the presence of 2.5 µM astaxanthin (red). (**B**) First time derivative of the action potential upstroke with the same color code. (**C**–**J**) Average results obtained from 7 cells in control and in the presence of astaxanthin (black, C: control; red, AX: astaxanthin; columns and bars: mean ± SEM; asterisks: significant differences are noted by * with *p* < 0.05).

Action potentials were recorded under steady-state conditions at 700 ms cycle length. After stabilization of action potential parameters in control (containing also 0.1% ethanol) the cells were exposed to 2.5 µM AX for 4–6 min. As presented in Figure 6, the duration of action potentials was significantly reduced by AX both at 50% and 90% levels of repolarization (APD_50_ and APD_90_, respectively, Figure 6A,D,F). AX also significantly decreased the maximal rate of depolarization (V^+^max) and the mid-plateau potential (measured at the time of 50% repolarization, Figure 6H,I). Other parameters, such as the resting membrane potential (−84.3 ± 0.9 vs. −83.1 ± 2.1 mV, Figure 6C), action potential amplitude (118.8 ± 2.4 vs. 113.5 ± 4.5 mV, Figure 6E), and maximal rate of repolarization (−1.4 ± 0.1 vs. −1.4 ± 0.1 V/s, Figure 6J) were not altered significantly by AX.

In conclusion, acute AX administration does not seem to alter significantly cardiac excitability thus no serious risk of arrhythmias can be predicted.

## 3. Discussion and Conclusions

In this work, AX supplementation did not change markedly the metabolic parameters measured in vivo, however it facilitated food intake. As a possible reason for changes in food intake, inhibitory inputs of hypothalamic POMC neurons were strengthened. In parallel with these, carbohydrate signaling of skeletal muscle was altered which potentially increased metabolism in muscle and caused anti-diabetic effects. Kinetics of the cardiac action potentials were slightly altered but this change did not predict a greater risk for arrhythmias.

### 3.1. In Vivo Effects of Astaxanthin

We propose that AX supplemented feeding could affect the glucose metabolism and fatty acid biosynthesis in mouse skeletal muscles. The cumulative food intake increased significantly during 4 weeks of treatment in the AX group (Figure 1E). The cumulative O_2_-usage and cumulative CO_2_-production were not altered significantly following 4 weeks of AX supplementation (Figure 1A,C). On the other hand, the XY-movements and total activity were significantly lower in each group following 4 weeks of normal or special AX feeding. These findings could explain why AX administration would not be enough to counteract the effects of weight gain by itself (Figure 1G) due to increased food consumption. We thus propose that AX administration should be combined with adequate diet regimen and exercise to maintain a healthy body mass index.

### 3.2. Effects of Astaxanthin on Skeletal Muscle Metabolism

Our data suggest that AX exerts new signaling effects which can greatly influence skeletal muscle metabolism.

We detected significantly altered mRNA, and in some cases (see below) protein expression levels of Fasn, Pdk4, Scd1, and Xdh in AX supplemented muscle samples compared to the control group. Studies have shown that the increase in muscle Pdk4 activity in starvation and streptozotocin-induced diabetes were due to a selective upregulation of Pdk4 expression [31], which may largely be due to insulin deficiency rather than to increases in circulating free fatty acids (FFA) [32,33,34]. Data also suggest that increased Pdk4 activity in skeletal muscle might contribute to the development of insulin resistance by suppressing glucose oxidation [31]. Insulin stimulation of PI3K and/or Akt is often impaired in insulin resistant states [35,36], and this would decrease insulin’s ability to regulate FOXO1 activity [37] and Pdk4 expression. Thus, increased Pdk4 expression, which would inhibit glucose oxidation, may be an important adaptive mechanism for glucose conservation [38]. Pdk4 phosphorylates and inhibits the PDH complex that catalyzes the conversion of pyruvate to acetyl-CoA. In our case, lower Pdk4 level and thereby increased PDH activity likely increased the generation of acetyl-CoA. This further elevated the synthesis of malonyl-CoA, the first molecule of fatty acid synthesis, causing increased Fasn4 production. Activation of PDH by downregulation of Pdk4 supports glucose catabolism instead of fatty acid utilization. Intracellular malonyl-CoA is an important factor in the regulation of fatty acids (FA); in healthy muscle, malonyl-CoA levels are associated with reciprocal changes in FA oxidation. Altogether, our results suggest increased glucose uptake which provides the possibility of elevated protein synthesis in skeletal muscles. Nevertheless, we recorded cumulative O_2_ usage, CO_2_ production, VO_2_, and VCO_2_ that were higher in AX animals, possibly because of their increased body weight. This explains why the normalized parameters were found to be significantly lower following AX supplemented feeding.

AS160 is a Rab GAP that regulates glucose uptake of skeletal muscle by controlling the activity of Rab GTPases. As a consequence, vesicular transport of GLUT4 from the cytosol to the plasma membrane increases which leads to increased glucose uptake. The increased phosphorylation of AS160 (pAS160) is an essential signal for the glucose uptake of skeletal muscle and adipose tissue. Earlier studies described that AX treatment increases glucose uptake in L6 myoblasts [19] or in high fat and high fructose diet-fed mice [20]. In our work, AX feeding significantly increased pAS160/AS160 levels in both biceps femoris and pectoralis muscle samples (Figure 2A,B and Figure S2). The increased phosphorylation of AS160 can explain the beneficial effects of AX to improve insulin sensitivity. In the case of insulin resistance and type-2 diabetes mellitus the cellular amount of GLUT4 is decreased [39] and its translocation is impaired [40]; therefore, the improvement of GLUT4 translocation decreases blood glucose level. The increased pAS160/AS160 ratio following AX feeding presumably increases intracellular glucose levels and, consequently energy levels, allowing cells to improve protein synthesis and muscle mass gain. This idea is further supported by an increase in mTOR phosphorylation in the AX-fed group, since mTOR stimulates protein synthesis and inhibits autophagy [41,42]. Here, we found that AX feeding increased mTOR phosphorylation (Ser2448) in biceps femoris and pectoralis muscles (Figure 2A,B and Figure S2). Since the total amount of mTOR also increased, the mTOR(pSer2448)/mTOR ratio did not change significantly following AX treatment.

PGC-1α is a transcriptional coactivator important in energy metabolism and a master regulator of mitochondrial biogenesis. Moreover, PGC-1α also increases GLUT4 levels and has multiple roles in the pathogenesis of type-2 diabetes mellitus [43,44]. In our experiments, the expression of PGC-1α slightly increased in biceps femoris and pectoralis muscle samples following AX administration (Figure 2A,B and Figure S2) which is in accordance with a previous work [12] where PGC-1α was significantly elevated in skeletal muscle samples following AX intake in mice.

Although increased phosphorylation of Akt was earlier described in L6 muscle cells following AX administration [19], the p(Ser474)Akt2/Akt2 ratio did not change in our study (Figure 2A,B and Figure S2).

### 3.3. Effects of Astaxanthin on Hypothalamic Neuronal Activity

The hypothalamic arcuate nucleus has a central role in food intake. Its two major neuronal types have distinct roles in appetite regulation: the anorexigenic POMC neurons decrease, whereas the orexigenic NPY- and AgRP-expressing neurons stimulate food intake [26,27,28]. Decreased activity of POMC cells is associated with obesity [26,29], whereas selective stimulation of them leads to weight loss [45].

GABAergic inputs to POMC neurons are capable of modulating them according to the actual energetic status of the organism [46]. POMC cells receive GABAergic inhibitory inputs from the dorsomedial hypothalamus (DMH) and the orexigenic hypothalamic AgRP neurons [26,29,47,48]. The frequency of spontaneous IPSCs onto POMC neurons increases during caloric deficits [26].

Glucose and fatty acid metabolism can affect cells of the arcuate nucleus by defining functional subgroups. There are glucose-excited and glucose-inhibited neurons reflecting changes of extracellular glucose levels in the normal range. Direct interaction was shown between glucose metabolism and fatty acid detection in regulation of neuronal activity in the arcuate nucleus. There are both oleic acid (OA) excited and oleic acid inhibited neurons, which are sensing glucose as well [49,50,51,52].

Mendoza et al. [53] investigated the effect of krill oil on restraint stress (RS) causing depressive-like behavior, memory deficiency and anxiety. Krill oil with high AX content has been shown to decrease depressive-like behavior and oxidative stress. Applying krill oil prevented appearance of depressive-like behavior and cognitive impairment by RS.

In the present work, we surprisingly found that, in contrast with oleic acid, AX did not elicit tonic changes of excitability. However, resembling conditions in hunger, an increase of sIPSC frequency on POMC neurons was seen. This increase was due to pleiotropic actions on GABAergic neurons, as the spontaneous firing rate was increased, probably partially caused by the strengthening of excitatory inputs in some cases. As inhibition of POMC neurons increases feeding, the increase of GABAergic activity might contribute to the increased food consumption seen with chronic AX supplementation. Although coronal slices contained the main sources of GABAergic inputs of the POMC neurons, the arcuate nucleus and the DMH, actions on the IPSCs were probably underestimated, as certain synaptic connections were lost in brain slices.

### 3.4. Effects of Astaxanthin on Cardiac Excitability

According to our knowledge cellular electrophysiological effects of AX in mammalian cardiac tissues were not reported yet. In present study, we used canine ventricular myocytes as their electrical properties greatly resemble those of the human myocardium [54,55]. Furthermore, canine heart is believed to be a good model to study the cardioprotective effects of AX [56]. Acute AX treatment shortened the action potential, depressed the plateau potential and reduced depolarization velocity (Figure 6A,I,H). These changes are congruent with a mild inhibition of inward currents (such as the L-type Ca^2+^ and Na^+^ currents) by AX. A reduction in Ca^2+^ entry may also contribute to the well documented cardioprotective effect of AX [57], presently attributed to decreasing the consequences of oxidative stress by supporting the function of the mitochondria [15,58,59]. Since the resting potential and the maximal rate of repolarization remained unchanged, AX seems to leave the inward rectifier K^+^ current unaffected (Figure 6C,J). However, further detailed studies are required to elucidate the exact molecular mechanisms of the cardiac electrophysiological effects of AX.

**Figure 7:**
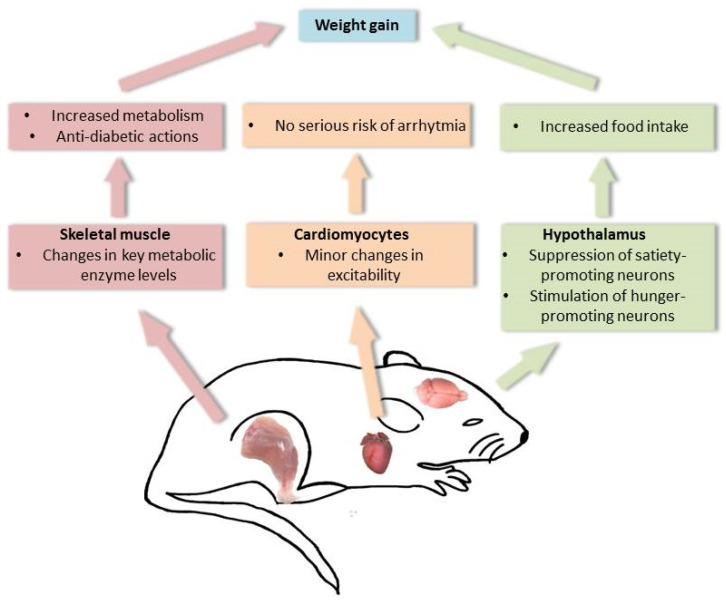
Proposed mode of action of AX in mice. Summary of our findings on the responses by AX on skeletal muscle metabolism, cardiac excitability and hypothalamic neuronal activity that could lead potentially to weight gain. AX facilitates the increase in metabolism and exerts anti-diabetic actions on skeletal muscle, promotes food intake via actions on the hypothalamus, and exerts minor changes on cardiac excitability.

In conclusion, we propose that AX exerts complex effects on whole body metabolism (Figure 7). It affects food intake via hypothalamic regulatory centers of energy homeostasis, as well as via increased metabolism and anti-diabetic actions by direct effects on the target organs of mice; without presenting serious risks for cardiac arrhythmias on a canine model. Our results support the idea that AX, this widely used nutraceutical, is a potent candidate for treatment of diseases of energy homeostasis; however, one must acknowledge that solely its administration will not replace a healthy diet and lifestyle but rather will be best achieved in conjunction with them.

## 4. Materials and Methods

### 4.1. Animals

Animal experiments were conducted in accordance with the appropriate national and international (EU Directive 2010/63/EU for animal experiments) institutional guidelines and laws on the care of research animals. Experimental protocols were approved by the Committee of Animal Research of the University of Debrecen (3-1/2019/DEMÁB).

Young adult (3-months-old) wild-type mice C57BL6 (*n* = 16) were used for chronic, 4-week-long AX feeding experiments (8 were fed with AX supplemented chow and 8 were fed normal rodent chow). The special chow was prepared with the addition of 4 g/kg of AstaReal A1010 (dissolved in 100% ethanol) to the standard rodent pellet (protein 20%, carbohydrates 70%, fats 4%, fibers 5%, vitamins, micro- and macronutrients) for a final concentration of 0.02% AX. This concentration was chosen according to the literature [13,15]. The mice had ad libitum access to water and food intake. Room illumination was set automatically to cycles of 12-h dark and 12-h light.

For acute experiments, mice expressing the tdTomato fluorescent protein in a proopiomelanocortin (POMC)- or glutamate decarboxylase type 2 (GAD2)-dependent way (*n* = 8 for both groups), as well as mice expressing GCaMP6f genetically encoded calcium indicator in a type 2 vesicular gamma-amino-butyric acid transporter (VGAT2) dependent way were used (*n* = 3) from both sexes. Homozygous floxed-stop tdTomato (B6;129S6-Gt(ROSA)26Sortm9(CAG-tdTomato)Hze/J; JAX mice accession number 007905) and GAD2-cre lines (STOCK Gad2tm2(cre)Zjh/J; JAX number: 010802), as well as the heterozygous POMC-cre line (STOCK Tg(Pomc1-cre)16Lowl/J; JAX number: 005965M) were purchased from Jackson Laboratories (Bar Harbor, ME, USA) and were crossed in the animal house of the Department of Physiology. Homozygous floxed-stop GCaMP6f (B6;129S-Gt(ROSA)26Sortm95.1(CAG-GCaMP6f)Hze/J; JAX number: 024105) and VGAT2-cre mice (STOCK Slc32a1tm2(cre)Lowl/J; JAX number: 016962) were also purchased from Jackson Laboratories and crossed in the animal house of the Department of Anatomy. VGAT2-GCaMP6 mice were generously provided by Dr. Péter Szücs (Department of Anatomy, University of Debrecen).

Adult mongrel dogs of either sex were anaesthetized with intramuscular injections of 10 mg/kg ketamine hydrochloride (Calypsol, Richter Gedeon, Budapest, Hungary) and 1 mg/kg xylazine hydrochloride (Sedaxylan, Eurovet Animal Health BV, Bladel, The Netherlands) according to protocols approved by the local ethical committee (9/2015/DEMÁB) in line with the ethical standards laid down in the Declaration of Helsinki.

### 4.2. Metabolic Cage Experiments

A Comprehensive Lab Animal Monitoring System (CLAMS) was used to monitor the metabolism of 8 control and 8 AX-treated mice. Three-month-old animals were selected for the experiments and were divided into 4 groups. As the monitoring system could measure 8 animals at the same time, 4–4 mice were measured for control and AX group, then the remaining 4–4 animals were put into the special cages. During 48 h of continuous monitoring, several parameters (food and water intake, calorimetry data, activity of the animals, and sleeping time) were followed where total or accelerated (cumulative) data were given for the whole measuring period or for 12 h of dark and light daily periods.

Animal activity was recorded with an array of infrared photo beams that surround the cage. When the animal moved, the beams were interrupted, generating one count. Since the beams were arranged in a grid pattern, the XY position of the animal could be continuously recorded (X_amb_). When equipped with the Z axis sensor, total movements could be determined (X_total_). Bouts of inactivity of certain duration were scored in an event file as sleeping events.

The system collected calorimetry parameters: VO_2_, VCO_2_, RER, and heat production. The calculation of oxygen consumption (VO_2_) and carbon dioxide production (VCO_2_) values required the use of both the input (Vi) and output (Vo) flows to the chamber. The respiratory exchange ratio (RER) can be calculated from either set of consumption and production rates (RER=VCO_2_/VO_2_). Calorific value (CV) was calculated from RER using the following equation: CV = 3.815 × 1.232 × RER. Total energy expenditure (TEE) was calculated by multiplying CV and VO_2_. As this parameter contain resting/basal metabolic rate and activity related energy expenditure, resting metabolic rate was determined by averaging the ten minima values of energy expenditure within the 48-h measurement. RMR was subtracted from TEE giving the TEE activity values.

### 4.3. Metabolic Pathways Panel

Functional annotations for different pathways and processes were assigned to the genes in the nCounter Metabolic Pathways Panel (Nanostring, Fairview, WA, USA). The pathways and processes that are included in this panel provide a comprehensive view of cell metabolism. Exact genes regarding the different metabolic pathways can be found at the website of Nanostring company.

Total RNA samples were prepared from m. extensor digitorum longus (EDL) of 6 control and 6 AX-treated mice following 4 weeks of special feeding. The samples were subjected to RNA isolation using Trizol reagent (Sigma–Aldrich, St. Louis, MO, USA). The concentration of the samples was determined by the Nanodrop2000 spectrophotometer (ThermoFisher Scientific, Waltham, MA, USA). Samples were loaded into the specific metabolic panel and the appropriate reaction was carried out. Data were first analyzed using the nSolver program, where all data from the metabolic program measurement were normalized to 12 different housekeeping genes and the threshold suggested by Nanostring was determined from these results. Significant differences between control and AX-treated samples were determined by giving the fold change ratio of the appropriate genes. BRB array tool analysis was used to further improve the quality of the measurement and to ensure the assessment of significant changes of certain genes.

### 4.4. Protein Isolation and Western Blotting

*EDL*, biceps femoris, and pectoralis muscles of control and AX-fed mice were homogenized in buffer containing 50 mM of Tris-HCl pH 7.6, 100 mM of NaCl, 10 mM of EDTA, 1 mM of NaF, 1 mM of Na_3_VO_4_, and protease inhibitor cocktail (Sigma–Aldrich, St. Louis, MO, USA). After centrifugation of the samples at 16,000× *g* for 10 min at 4 °C to eliminate cellular debris, the protein concentration of the supernatants was measured using BCA Protein Assay Kit (Thermo Fisher Scientific, Waltham, MA, USA). The samples (25 μg/lane) were separated by SDS/PAGE, and blotted to Protran nitrocellulose membrane (Amersham, GE Healthcare, Little Chalfont, UK). After blocking, membranes were overnight incubated with rabbit anti-phospho(Ser2448)-mTOR (#5536), mTOR (#2983), phospho(Thr642)-AS160 (#8881), AS160 (#2670), PGC-1α (#2178), phospho(Ser474)-Akt2 (#8599), Akt2 (#3063), or mouse anti-GAPDH (#2118) primary antibodies, all from Cell Signaling Technology (Danvers, MA, USA). This was followed by incubation with the appropriate horse-radish peroxidase-conjugated anti-IgG secondary antibodies from DAKO (Glostrup, Denmark). In other experiments membranes were incubated with mouse anti-FASN, mouse anti-SCD1, and rabbit anti-PDK4 primary antibodies (all from Novus Biologicals, Abingdon, UK), followed by incubation with appropriate HRP-conjugated secondary antibodies (Bio-Rad, Hercules, CA, USA). Peroxidase activity was visualized by the enhanced chemiluminescent procedure (Advansta, San Jose, CA, USA; and Thermo Fisher, Waltham, MA, USA). Quantification of signal intensity was performed by Quantity One software (Bio-Rad, Hercules, CA, USA) and ImageJ (NIH, Bethesda, MD, USA).

### 4.5. Acute Hypothalamic Slice Preparation

The recording solution for experiments on the hypothalamus was an artificial cerebrospinal fluid (aCSF) with the following composition (in mM): NaCl, 120; KCl, 2.5; NaHCO3, 26; glucose, 10; NaH2PO4, 1.25; CaCl2, 2; MgCl2, 1; myo-inositol, 3; ascorbic acid, 0.5; and sodium-pyruvate, 2; pH 7.2. In the solution used for preparation (low Na aCSF), 95 mM of NaCl was replaced by glycerol (60 mM) and sucrose (130 mM). All chemicals were purchased from Sigma (St. Louis, MO, USA) unless stated otherwise.

After decapitation of the animal and removal of the brain, 200-μm-thick coronal slices were prepared from the area including the hypothalamus in ice-cold low Na aCSF with a Microm HM 650V vibratome (Microm International GmbH, Walldorf, Germany). Prior to recording, the slices were incubated in normal aCSF for 60 min at 37 °C.

### 4.6. Isolation of Canine Ventricular Myocytes

Single myocytes were obtained by enzymatic dispersion using the segment perfusion technique. Briefly, the heart was quickly removed and washed in a cold Tyrode’s solution. Left anterior descending coronary artery was cannulated, then a wedge-shaped section of the ventricular wall supplied by the artery was dissected and perfused with a nominally Ca^2+^-free Joklik solution (Minimum Essential Medium Eagle, Joklik Modification, Sigma–Aldrich Co., St. Louis, MO, USA, product no. M0518) for 5 min. This was followed by perfusion with Joklik solution supplemented with 1 mg/mL collagenase (Type II, Worthington Biochemical Co., Lakewood, NJ, USA) and 0.2% bovine serum albumin (Fraction V., Sigma) containing 50 µM Ca^2+^ for 30 min. After this, normal external Ca^2+^ concentration was gradually restored, and cells were kept in Minimum Essential Medium Eagle (Sigma–Aldrich Co., product no. M0643) until use.

### 4.7. Electrophysiology

Hypothalamic coronal slices were visualized with a Zeiss Axioscope microscope (Carl Zeiss AG, Oberkochen, Germany). Patch pipettes with 6–7-MΩ pipette resistance were pulled, and filled with internal solution composed of (in mM): K-gluconate, 120; NaCl, 5; 4-(2-hydroxyethyl)-1- piperazineethanesulfonic acid (HEPES), 10; EGTA, 2; CaCl_2_, 0.1; Mg-ATP, 5; Na3-GTP, 0.3; Na2- phosphocreatine, 10; biocytin, 8; pH 7.3. Whole-cell patch-clamp recordings were performed using an Axopatch 200A amplifier (Molecular Devices, Union City, CA, USA). Data acquisition was achieved by Clampex 10.0 software (Molecular Devices, Union City, CA, USA), while data analysis was performed using Clampfit 10.0 (Molecular Devices) and MiniAnalysis (Synaptosoft, Decatur, GA, USA) software. During recording, slices were kept in oxygenated nACSF at room temperature. For testing acute effects of AX, 2.5 µM AX was dissolved in nACSF with 0.1% ethanol. In preliminary experiments on spontaneous EPSCs and IPSCs, the action of aCSF with 0.1% ethanol was compared with ethanol-free nACSF and no significant changes were found.

For recording action potential trains, a current clamp protocol with 1-s-long depolarizing steps was employed from −30 to +120 pA with 10 pA increments. The resting membrane potential was set to −60 mV. Recordings of spontaneous firing rates was done in current clamp mode. The neurons were kept on their own resting membrane potential and no current injection was administered. Fifty-second-long traces were evaluated with each neuron and condition.

For tonic currents and spontaneous EPSCs and IPSCs, gap-free voltage-clamp traces were recorded at a holding potential of −60 mV before and after drug application (10 min in each condition). Tonic currents were assessed by making histograms of the recorded current values from the last minute of the trace; and the current values at the histogram peaks were considered as the amplitude of the tonic current. Spontaneous changes of the holding current were calculated as the difference between the first and the last minute of the control recording. At the end of the experiment, slices were continuously perfused with 10 μM of 2,3–dihydroxy–6–nitro–7–sulfamoyl-benzo[f]quinoxaline–2,3–dione (NBQX), 50 μM if D–2–amino–5–phosphonopentanoate (D-AP5), 1 μM of strychnine, and 10 μM of bicuculline (Tocris Cookson Ltd., Bristol, UK).

Cardiac action potentials were recorded at 37 °C, maintained by an electronic temperature controller (Cell MicroControls, Norfolk, VA, USA). The rod-shaped viable cells showing clear striation were sedimented in a plexiglass chamber of 1-mL volume allowing continuous superfusion (at a rate of 2 mL/min) with Tyrode’s solution. The Tyrode solution contained (in mM): NaCl, 121; KCl, 4; MgCl_2_, 1; CaCl_2_, 1.3; HEPES, 10; glucose, 10; NaHCO_3_, 25; at pH = 7.3. Membrane voltage was recorded using 3 M of KCl filled sharp glass microelectrodes having tip resistance between 20 and 40 MΩ. These electrodes were connected to the input of an Axoclamp 2B, Multiclamp 700A or 700B amplifiers (Molecular Devices, Sunnyvale, CA, USA). The cells were paced through the recording electrode at steady cycle length of 0.7 s using 1–2 ms wide rectangular current pulses having amplitudes of 120% diastolic threshold. Action potentials were digitized (at 50 kHz using Digidata 1322A or 1440A A/D card, Molecular Devices, Sunnyvale, CA, USA) and stored for later analysis. Cells were exposed to 2.5 µM of AX for 4–6 min (dissolved in ethanol yielding a final ethanol concentration of 0.1%). Control solution also contained this concentration of ethanol.

### 4.8. Calcium Imaging

Calcium imaging experiments were performed on slices from VGAT2-GCaMP6 mice. Slices were prepared similarly as for electrophysiology experiments. A Zeiss Axioscope microscope (Carl Zeiss AG) equipped with a fluorescent imaging system (Till Photonics GmbH, Gräfeling, Germany) containing a xenon bulb-based Polychrome V light source, a CCD camera (SensiCam, PCO AG, Kelheim, Germany), an imaging control unit (ICU), and the Till Vision software (version 4.0.1.3) were used. The fluorescent filter set contained an emission filter (LP 515, Till Photonics) and a dichroic mirror (Omega XF2031 505DRLPXR; Omega Drive, Brattleboro, VT, USA). Frames with 344 × 260-pixel resolution were taken with a frame rate of 10 Hz.

### 4.9. Post Hoc Identification of the Labeled Neurons

During recording, neurons were labelled with biocytin. Slices with the filled neurons were fixed overnight (4% paraformaldehyde in 0.1 M of phosphate buffer; pH = 7.4; 4 °C). For permeabilization, Tris buffered saline (in mM, Tris base, 8; Trisma HCl, 42; NaCl, 150; pH = 7.4) with 0.1% Triton X-100 and 10% bovine serum was used for 60 min. Incubation was performed in phosphate buffer supplemented with streptavidin-conjugated Alexa488 (1:300; Molecular Probes Inc., Eugene, OR, USA) for 90 min. The cells were visualized with a Zeiss LSM 510 confocal microscope (Carl Zeiss AG).

### 4.10. Statistics

Results are expressed as mean ± SEM values. GraphPad Prism 7.0 (GraphPad Software Inc., San Diego, CA, USA) was used for graphing and statistical analyses. Statistical significance of differences was evaluated using Student’s *t*-test. Differences were considered significant when *p* was less than 0.05.

## Data Availability

Data will be made available on reasonable request.

## Supplementary Materials

The following supporting information can be downloaded at: https://www.mdpi.com/article/10.3390/ijms23020917/s1.
