# Supplementary material for: Astaxanthin Exerts Anabolic Effects via Pleiotropic Modulation of the Excitable Tissue
Source: Int J Mol Sci. 2022 Jan 14;23(2):917. doi: 10.3390/ijms23020917 (PMC8778848; doi:10.3390/ijms23020917)
Supplement: Supplementary file 1 [file ijms-23-00917-s001.zip › ijms-1491391-supplementary.pdf]

Supplementary Materials

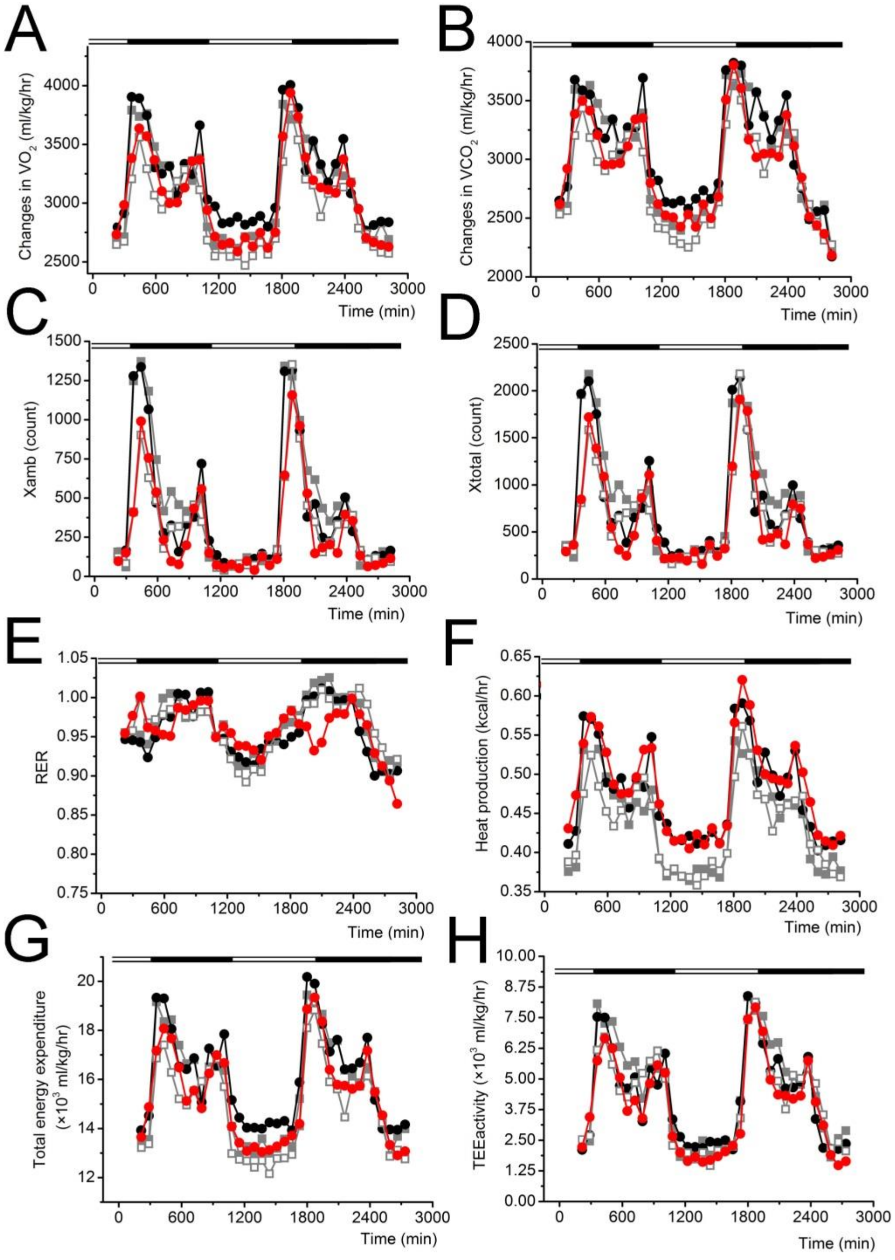

Figure S1. Changes of in vivo metabolic parameters before and following astaxanthin feeding. Continuous changes of VO_2 (**A**), VCO_2 (**B**), Xamb (**C**), Xtotal (**D**), RER (**E**), and heat production (**F**). Data from 4 measured points (equal with 4 × 18 minutes) were averaged for all animals and time course of the aforementioned parameters were plotted as a function of time. Total energy expenditure (TEE) was calculated by multiplying CV and VO_2 and plotted as a function of time (**G**). As this parameter contain resting/basal metabolic rate and activity related energy expenditure, we determined resting metabolic rate by averaging the ten minima values of energy expenditure within the 48-hour measurement. RMR was subtracted from TEE giving the $TEE_{activity}$ values of the different animal groups (**H**). Black lines above the graphs indicate periods in darkness, whereas hollow lines represent periods of light.

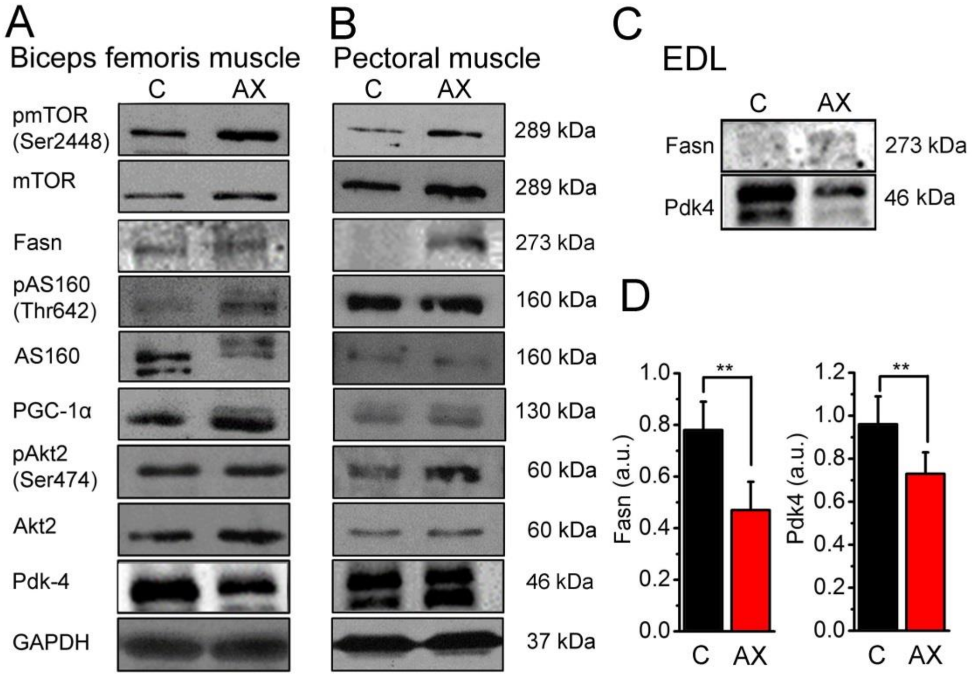

Figure S2. Effects of astaxanthin feeding on the levels of signaling proteins in biceps femoris, pectoral and extensor digitorum longus muscles. (**A**) Representative Western blot images show the levels of phospho(Ser2448)-mTOR, mTOR, Fasn, phospho(Thr642)-AS160, AS160, PGC-1α, phospho(Ser474)-Akt2, Akt2 and Pdk-4 in control samples (C) and in astaxanthin-treated mice (AX). GAPDH was used as a loading control. (**B**) Representative Western blot images from samples obtained from pectoralis muscles show the levels of phospho(Ser2448)-mTOR, mTOR, Fasn, phospho(Thr642)-AS160, AS160, PGC-1α, phospho(Ser474)-Akt2, Akt2 and Pdk4 in control samples (C) and AX treated mice, respectively. GAPDH was used as a loading control. (**C**) Representative Western blot experiments from extensor digitorum longus (EDL) muscle show significantly decreased Fasn and Pdk4 protein levels. (**D**) Statistical analysis of changes in Fasn and Pdk4 in EDL (black, C: control; red, AX: astaxanthin).

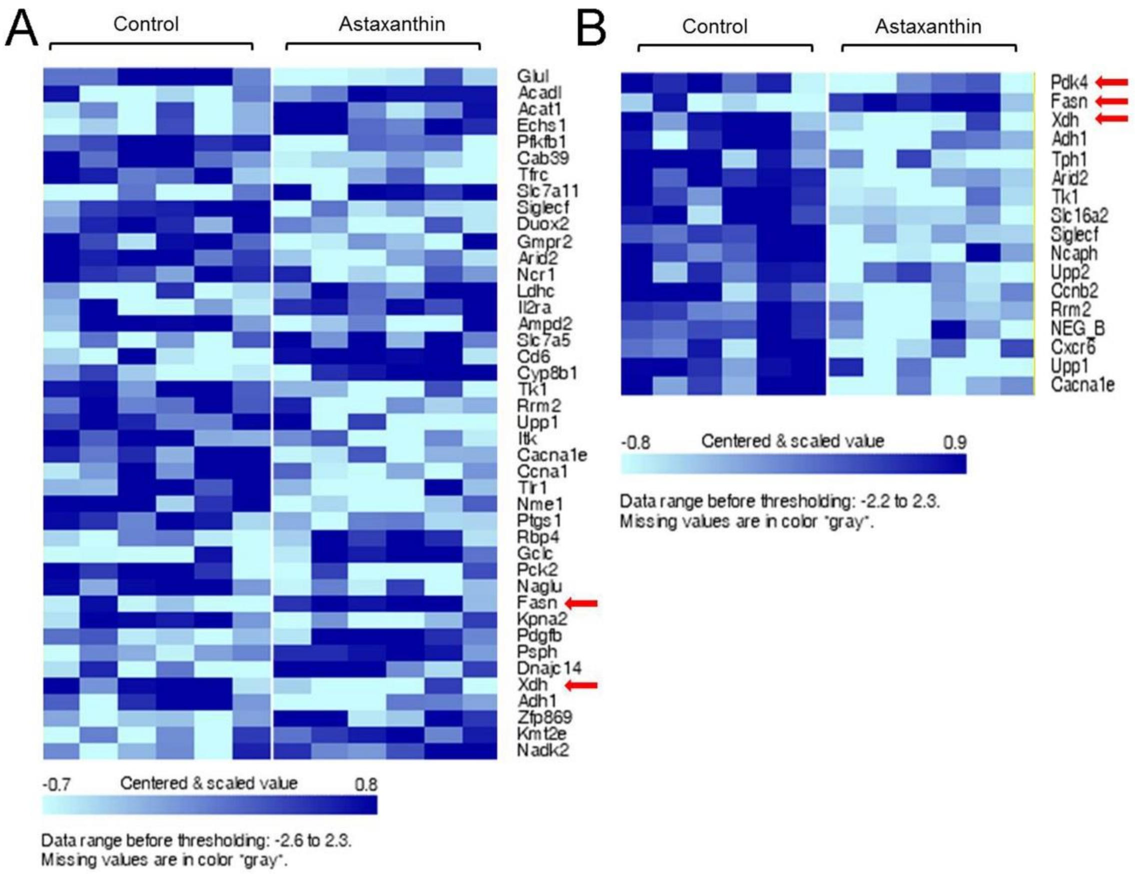

Figure S3. Clustered heatmap of significantly expressed genes in samples from EDL muscles. (**A)** Clustered heatmap following quantile normalization; arrays grouped by class. (**B)** Clustered heatmap of significantly expressed genes normalized to housekeeping genes; arrays grouped by class. Arrows indicate altered gene expression in AX treated samples compared to the controls.

Table S1. Metabolic parameters measured during 48 h before and after the 4 weeks of special feeding in control and astaxanthin-treated mice. Significance was calculated between control and astaxanthin-treated groups at different periods of the day and accepted to be significantly different, when $p < 0.05$.

		1st Period (Dark)		2nd Period (Light)		3rd Period (Dark)		4th Period (Light)	
		**Ctrl**	**Astx**	**Ctrl**	**Astx**	**Ctrl**	**Astx**	**Ctrl**	**Astx**
VO_2 (ml/kg/hr)	Before	3395.9 ± 32.1	3430 ±32.3	2747.8±28.5	**2927.9±28.6**	3383.5±32.91	**3466.5±32.5**	2735.2±37.4	2816.3±38.1
	After	3208.2±28.9	**3291.2±29.9**	2588.2±24.5	**2699.9±23.5**	3244.6±31.3	**3369.9±30.9**	2660.2±34.7	2711.2±33.9
VCO_2 (ml/kg/hr)	Before	3333.4±32.9	3350.6±33.1	2584.9±31.0	**2746.6±30.6**	3386.9±35.2	3422.3±33.7	2468.2±49.2	2463.9±53.9
	After	3144.0±30.2	**3218.9±31.3**	2412.5±27.7	**2562.2±24.9**	3226.3±32.7	3271.7±32.9	2438.6±45.6	2423.6±44.3
X_{amb} (counts)	Before	692.1±37.1	**594.3±35.5**	133.9±16.9	156.5±18.2	656.9±37.6	**563.2±33.9**	116.6±20.1	131.0±18.5
	After	440.5±26.2	428.3±27.8	93.2±9.3	84.4±8.4	509.7±32.9	472.3±29.3	108.7±13.8	88.5±12.3
X_{total} (counts)	Before	1198.8±53.2	**1049.3±50.9**	326.5±27.1	377.9±29.3	1164.1±54.4	**1024.6±46.9**	292.6±35.3	312.6±33.6
	After	876.6±40.7	858.4±43.9	267.8±17.9	264.8±16.7	960.2±49.0	924.7±45.6	287.7±26.0	263.6±25.1
RER	Before	0.92±0.003	0.97±0.003	0.94±0.004	0.93±0.003	0.99±0.002	**0.98±0.002**	0.92±0.005	0.9±0.005
	After	0.98±0.003	0.97±0.003	0.93±0.004	0.95±0.003	0.99±0.002	0.97±0.003	0.93±0.004	0.91±0.004
Heat production (kcal/hr)	Before	0.48±.0.005	**0.51±0.005**	0.38±0.004	**0.43±0.005**	0.48±0.005	**0.52±0.005**	0.38±0.006	**0.41±0.006**
	After	0.47±0.004	**0.52±0.005**	0.38±0.004	**0.42±0.004**	0.48±0.005	**0.53±0.005**	0.39±0.005	**0.42±0.005**
Total sleep time (min)	Before			156.0±8.4	137.8±15.4	51.6±7.5	54.8±9.8	207.4±14.8	192.4±21.1
	After			152.1±15.1	137.5±11.7	75.4±10.7	55.1±7.5	234.8±25.3	192.4±13.4
